# The Effect of a Pilot Dietary Intervention on Pain Outcomes in Patients Attending a Tertiary Pain Service

**DOI:** 10.3390/nu11010181

**Published:** 2019-01-16

**Authors:** Katherine Brain, Tracy L. Burrows, Megan E. Rollo, Chris Hayes, Fiona J. Hodson, Clare E. Collins

**Affiliations:** 1 School Health Science, Faculty of Health and Medicine, University of Newcastle, Callaghan, NSW 2308, Australia; katherine.brain@uon.edu.au (K.B.); Tracy.Burrows@newcastle.edu.au (T.L.B.); Megan.Rollo@newcastle.edu.au (M.E.R.); 2 Priority Research Centre in Physical Activity and Nutrition, University of Newcastle, Callaghan, NSW 2308, Australia; 3 Hunter Integrated Pain Service, Newcastle, NSW 2300, Australia; Chris.Hayes@hnehealth.nsw.gov.au (C.H.); Fiona.Hodson@health.nsw.gov.au (F.J.H.)

**Keywords:** chronic pain, dietary intervention, pain, quality of life, polyphenols, anthocyanins

## Abstract

The aim of this study was to examine the effect of a six-week 2 × 2 design on pain scores, quality of life, and dietary intake in patients attending an Australian tertiary pain clinic. The two intervention components were (1) personalized dietary consultations or waitlist control, and (2) active or placebo dietary supplement (fruit juice). Sixty participants were randomized into one of four groups at baseline (68% female, mean age 49 ± 15 years) with 42 completing the study (70% retention). All groups had statistically significant improvements in three of five pain outcomes. The personalized dietary consultation groups had clinically important improvements in three of five pain outcomes compared to the waitlist control groups. All groups had a statistically significant improvement in six of eight quality-of-life categories post intervention. All groups increased percentage energy from nutrient-dense foods (+5.2 ± 1.4%, *p* < 0.001) with a significant group-by-time effect for percentage energy from total fat (*p* = 0.024), with the personalized dietary consultations plus placebo fruit juice reporting the largest reduction (−5.7 ± 2.3%). This study indicates that dietitian-delivered dietary intervention can improve pain scores, quality of life, and dietary intake of people experiencing chronic pain. Future research should evaluate efficacy in a full-powered randomized control trial.

## 1. Introduction

Chronic non-cancer pain is pain that persists beyond the three months that it normally takes for tissues to heal and is not due to active cancer [1]. Chronic non-cancer pain, also termed “chronic pain” has many triggers including injury or disease. However, there is no obvious physical cause in about one-third of cases [2]. Despite numerous catalysts for chronic pain, a common pathophysiological explanation relates to hypersensitivity of the nervous system and associated dysfunction of the immune and endocrine systems [3,4]. One in five people aged 18 years and over experience chronic pain and this increases to one in three people aged 65 years and over [2]. Many people who experience chronic pain have a poor quality of life, as chronic pain is associated with depression, social isolation, and limited mobility [5]. There is also a significant economic burden with total costs due to chronic pain in Australia in 2007 estimated at $34 billion [2]. This includes $11.7 billion in productivity costs, $11.5 billion in burden of disease, and $7 billion in healthcare system costs [2].

With a strong bidirectional link between dietary intake and chronic pain experiences, investigation into the role of nutrition in chronic pain management is of growing interest to researchers and clinicians [6]. The individual experience of chronic pain can lead to poor appetite and sub-optimal dietary intake [7,8] and adversely impact ability to shop for food and cook meals. People who experience chronic pain may rely heavily on convenience and fast foods which are easier to prepare; however, these are often energy-dense and nutrient-poor [7]. An added complexity is the emotional response to chronic pain which can lead to contrasting responses from complete disinterest in food or the use of food and beverages as a comfort measure with subsequent overconsumption [9]. A qualitative study exploring the experiences of adults (87% aged >50 years) who have chronic pain and a body mass index (BMI) ≥25 kg/m^2^ found that emotional or binge-eating behaviors as a response to chronic pain were reported commonly, and coincided with depression and negative feelings such as guilt [9]. Equally important is the impact of dietary intake on the chronic pain experience itself. Diets which are low in fruit and vegetables and high in refined or ultra-processed foods, indicative of the typical “Western diet” contribute to a pro-inflammatory state associated with worsening of the chronic pain experience [10,11]. A systematic review of 71 experimental studies investigated the impact of altering dietary patterns, single nutrients, dietary supplements, or fasting on chronic pain experiences [12]. A meta-analysis identified that altering dietary intake led to a weighted mean reduction in self-reported pain scores (0.9 cm (0.54 cm, 1.27 cm)) in studies which used a visual analog scale to measure pain [12]. Existing studies in nutrition and chronic pain were not high quality, with half of the studies included in the review rated as of neutral or low quality using a standardized risk of bias tool, mainly due to interventions not being well described or not detailed enough to allow replication [12]. 

While the systematic review identified that prescribing a healthy diet assists in pain reduction, there are still a number of complexities and barriers (e.g., limited mobility affecting food preparation and/or limited motivation to change behavior) which need to be considered in population groups experiencing chronic pain, with research examining personalized dietary interventions needed to address these issues and identify appropriate and effective treatment options. The effectiveness of an intervention is also dependent on individuals’ behaviors, and changing this behavior is often difficult. The likelihood of an intervention to be effective and successful can be improved using evidence-based principles of behavior change theories. Theories such as the Behavior Change Wheel conceptualize aspects which influence the behavior of individuals so that they can be incorporated into interventions [13]. The Behavior Change Wheel incorporates three concepts which influence behavior change: capability (psychological and physical ability to engage in an activity), motivation (the brain’s ability to encourage or direct behavior beyond goals and conscious decision-making), and opportunity (factors that lie outside the control of the individual) [13]. In identifying any gaps individuals may have in these three concepts, researchers can tailor interventions to increase the capability, motivation, and opportunity for those involved and help promote overall behavior change [13].

Emerging evidence supports a potential role for non-nutritive bioactive compounds in reducing inflammation and modulating the chronic pain experience [14,15]. Polyphenol is an umbrella term for plant-based compounds which contain a polyphenolic substructure [16]. These can be further categorized into flavonoids and anthocyanins [16]. Anthocyanins are water-soluble pigments responsible for the red, purple, and blue colors in food, and the main type of anthocyanin found in plants is called cyanidin [17]. Of the edible plants containing anthocyanins, cherries were identified as containing high concentrations of anthocyanins and were used in in vitro [18,19,20], animal [21,22,23], and human studies [24,25] with characteristic metabolic impacts, including cardio and neuroprotective effects, anti-inflammatory action, and pain-modulating effect. Foods high in polyphenols, including cherries, strawberries, blueberries, and plums, were used in clinical studies [24,25,26,27]. In addition to potential antioxidant properties, mechanisms through which cherry anthocyanins act on inflammation and pain modulation include the inhibition of cyclooxygenases 1 and 2 (COX-1 and COX-2) [18,28]. Studies showed that anthocyanins are comparable to nonsteroidal anti-inflammatory drugs in terms of their ability to inhibit COX-1 and COX-2 enzyme activity [18,28]. The effect of anthocyanins on cognition were also explored in a randomized control trial where apple juice was used as the control fruit juice [29]. This current study also uses apple juice as the control fruit juice, given its low anthocyanin content [30].

Telehealth is being used increasingly in clinical services, providing greater access to health services for the community. A systematic review identified that using telehealth to provide dietary advice to adults with chronic disease is effective when compared to usual care, low-intensity in person dietary education or non-dietary interventions, in improving diet quality, with a standardized mean difference 0.22 (95% confidence intervals (CIs): 0.09, 0.34; *p* = 0.0007) and consumption of fruit and vegetables with a mean difference of 1.04 servings/day (95% CIs: 0.46, 1.62 servings/day; *p* = 0.0004) [31]. Guidelines for dietetic video consultations [32] for weight management were also developed that incorporate both telemedicine standards and nutrition care process [33,34].

Given pain is a subjective experience, it is also important to measure clinically important changes to evaluate whether patients obtain a meaningful change in their pain outcomes. Statistically significant results may not also mean clinically important results and vice versa, and that is why it is important to present clinically meaningful changes in pain outcomes [35,36]. The importance of measuring and interpreting clinically meaningful changes resulted in the development of recommendations for measuring clinically important changes set by the Initiative on Methods, Measurement, and Pain Assessment in Clinical Trials (IMMPACT) [37].

The aim of the current pilot study was to investigate the significance and clinical impact of two intervention components: (1) personalized dietary behavior change delivered using dietetic consultations or a waitlist control, and (2) the effects of an active or placebo dietary supplement, comprising either a high-anthocyanin fruit juice (cherry juice) or a placebo fruit juice with low levels of anthocyanins and antioxidants (reconstituted apple juice) on pain scores, quality of life, and dietary intake in patients attending a tertiary pain clinic. It was hypothesized that participants who received the dietary behavior change component and high-anthocyanin fruit juice would have a greater reduction in pain score compared to those randomized to the waitlist control plus the placebo juice group.

## 2. Materials and Methods

### 2.1. Participants

Participants were adults (≥18 years old) experiencing chronic pain and being treated by Hunter Integrated Pain Service (HIPS), New South Wales, Australia. HIPS is a multidisciplinary tertiary pain service, available to the public, by referral from a general practitioner or medical specialist, to people living in the Hunter New England Local Health District, and employs 16 clinicians and administrative staff, each with fractional appointments. Each year, over 1000 patients are referred to the service. HIPS uses a standardized group treatment pathway which includes a series of educative seminars and group workshops to promote a holistic and self-management approach to chronic pain management. Individualized assessment and treatment is offered in selected cases. The standardized pathway includes an orientation seminar, assessment workshop, treatment program, and a refresher workshop. These are called Understanding Pain (UP), Assessment and Planning (A&P), Active Pain Treatment (APT), and Progress Review Group (PRG).

### 2.2. Consent and Ethics

All participants were provided with an information statement and gave their informed consent for inclusion before they participated in the study. This study was conducted in accordance with the Declaration of Helsinki, and the protocol was approved by the Hunter New England Human Research Ethics Committee (17/07/19/4.04) and the University of Newcastle Human Research Ethics Committee (H-2017-0295). This trial was registered retrospectively with the Australian New Zealand Clinical Trials Registry (ACTRN12618001941257). 

### 2.3. Recruitment and Screening

**Figure 1:**
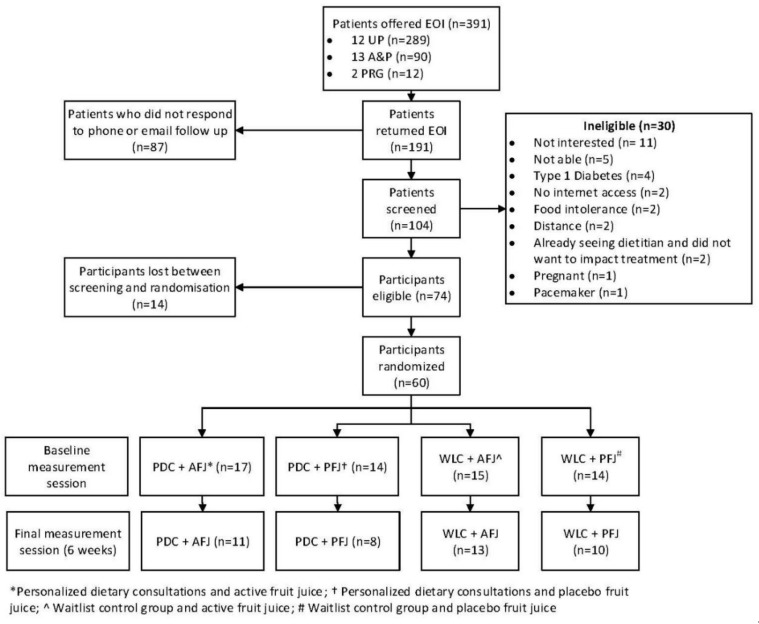
Participant flow through study.

Participants were recruited to the study if they attended either UP, A&P, or PRG between September 2017 and April 2018. Patients were not recruited from APT to prevent the treatment confounding the results of this study. The approximate wait between each session described above was three months and provided a sufficient window of time to ensure that the standard clinical care did not confound this study. All patients in each of these groups were offered an expression-of-interest form by the researcher (Figure 1). Either the student researcher or a HIPS clinician (when the student researcher was not available) provided a standardized two-minute verbal and visual explanation of the study which was presented at the end of each session, and information statements were made available to patients. Expression-of-interest forms were collected by the student researcher or clinician, and those who indicated interest in the study were then contacted via email and/or phone by the researcher. Patients were screened either via return email or phone interview. Patients were eligible if they had access to reliable broadband internet, were able to attend two in-person measurement sessions at the University of Newcastle, and were willing to provide a fasting blood sample. Patients were excluded if they had an intolerance to fruit, were pregnant, had a pacemaker or cochlear implant, and/or had a severe medical condition (e.g., insulin-controlled diabetes).

### 2.4. Intervention Inclusions and Delivery

The intervention comprised two components: (1) a dietary behavior change component delivering personalized dietary consultations (PDC) or waitlist control (WLC), and (2) a dietary supplement in the form of a fruit juice. There were two fruit juices included: (1) active fruit juice (AFJ) (cherry juice), and (2) placebo fruit juice (PFJ) (apple juice). The intervention ran for six weeks and included four study arms, which were provided with different combinations of the intervention components. These included (1) PDC and AFJ, (2) PDC and PFJ, (3) WLC and AFJ, and (4) WLC and PFJ. After participants were screened and completed the baseline assessment, they were stratified by gender and randomized, using a computer generated randomization tool, into one of the four study arms (Figure 1).

#### 2.4.1. Personalized Dietary Consultations

Participants received up to three personalized dietary consultations with an accredited practicing dietitian (APD) which were conducted using telehealth. The initial consultation was booked in the first week of the study, and the subsequent consultations were booked about 7–10 days after each other. Participants were encouraged to use the Avaya Scopia^®^ [38] (a video call platform used by Hunter New England Local Health District) to conduct the consultations. Participants were also given the option to conduct the consultation via a phone call. 

The participants received a copy of their Australian Eating Survey Report (AES) [39] at least 24 h prior to the first consultation, allowing them to become familiar with the contents. The AES Report was generated from the AES food frequency questionnaire completed at the measurement session. The AES is a valid and reliable online food frequency questionnaire which assesses participants’ usual food and nutrient intake over the past 3–6 months [39]. It takes approximately 15 minutes to complete and generates a personalized report which compares the participant’s intake to national nutrition guidelines. The report provides a pictorial representation of energy contributions from major food groups, breakdown of energy coming from macronutrients, and core and energy-dense, nutrient-poor food groups, compares micronutrient intake to the nutrient reference values [40], and calculates the Australian recommended food score which indicates overall diet quality [39].

Participants were also asked to complete a personalized nutrition questionnaire (PNQ) to guide discussion on perceived barriers during the call with the dietitian. The PNQ incorporates the Capability, Opportunity, Motivation Behavior (COM-B) model and the Behavior Change Wheel theory [13] in relation to factors which may affect eating behaviors, and it was tailored for patients experiencing chronic pain. When completing the PNQ, participants were asked to select and prioritize from a list of known factors which most affect their ability to eat healthily. These were presented in three categories: (1) capability: knowledge, skills, and ability; (2) opportunity: time, access, and storage; (3) motivation: wants, needs, and habits. The PNQ provided the dietitian with information on barriers prior to the initial consultation with the participant, allowed streamlining of the session, and facilitated the collection of additional information on the prioritized barriers during the consultation. Accompanying the PNQ was a toolbox where intervention strategies and resources were linked with each of these factors and tailored for individuals with chronic pain. Depending on which factors were selected by participants, the corresponding evidence-based resources and strategies were provided to help participants achieve their goals. The strategies which corresponded to these factors were based on the COM-B model and included, but were not limited to, education, instruction on how to perform a behavior, empowerment, problem solving, self-monitoring, and restructuring the environment. The resources included websites and handouts which were sourced from government departments, the Dietitians Association of Australia, Nutrition Education Materials Online, Australian Healthy Food Guide, and Practice-Based Evidence in Nutrition.

The first telehealth consultation (30–45 min) was structured as follows: introduction; explanation of the AES report with focus on the four main sections: food groups responsible for energy intake, the ARFS, macronutrients, and micronutrients; education about the food groups and nutrients important for chronic pain management (e.g., vegetables, fruits, antioxidants, omega-3, and vitamin B); discussion of the chosen PNQ priorities, setting goals, and discussing strategies and resources to achieve these goals. A summary of the consultation was emailed to each participant immediately after the consultation, and a second consultation was scheduled for 7–14 days later. The second consultation (≤30 min) included answering participant questions, identifying and discussing successes and barriers toward achieving goals, and troubleshooting solutions to any barriers. If necessary, additional resources were emailed to participants at the end of the consultation. The third consultation was optional and was limited to ≤30 min. This consultation focused on reinforcing education and strategies provided in the first two consults and an additional discussion around goals. 

#### 2.4.2. Dietary Supplement (Active Fruit Juice)

Cherry juice was chosen as the active fruit juice, for its high anthocyanin content (Table S1). The cherry juice was purchased from an agricultural research company (Agritechnology) based in Orange, New South Wales (NSW), Australia. Agritechnology produces the cherry juice with the aim to retain the phenolic bioactives. Total red count (TRC) is a measure for total anthocyanins; for the juice used in this pilot study, the TRC was 19.3 mg/100 g (Table S1). Typically, Agritechnology cherry juice is approximately 30 mg/100 g and can reach >100 mg/100 g depending on the season. Data from the Phenol-Explorer database [30] provides the content of anthocyanins and total polyphenols in various foods, and shows the high content of these in cherries. The anthocyanin content of cherries ranges from 54.3–171.42 mg/100 g, and the total polyphenol content ranges from 96.81–274.3 mg/100 g depending on the type of cherry [30]. Each participant was given 42 × 250 mL bottles of cherry juice at their baseline measurement session. Participants were given instructions to consume one bottle a day for six weeks, and advised that the juice should be stored in the refrigerator or in a dark and cool location until ready to consume. A written calendar was provided to participants as a reminder to consume the juice each day. Participants were asked to tick off each day once they consumed the juice, and to return the calendar at the second measurement session to assess compliance with the intervention. In an attempt to blind the study, participants were told there were two fruit juices, one active and one placebo, and that they would be randomly allocated to one or the other.

#### 2.4.3. Control Group Conditions

The control condition for the dietary behavior change component was a waitlist control group. This group was instructed to continue with their usual diet and not make any dietary changes. At the end of the six-week period, participants in this group were given the opportunity to participate in the dietary behavior change intervention and given full access to all components outlined above. 

The literature indicates that there are health benefits such as reduced risk of cancer, heart disease, asthma, and type 2 diabetes from consuming apple flavonoids (dihydrochalcones and flavanols) [30,41]. However, the reconstituted apple juice was processed in such a way that likely degraded any antioxidant content [42]. In addition, the Phenol-Explorer database shows that apples only have 0.93 mg of anthocyanins per 100 g and 56.32 mg of total polyphenols per 100 g [30]. Participants who received the apple juice were given the same quantity, instructions, and calendar to record their consumption. The apple juice was the Orchy brand, and was purchased from Bevco, based in Thornlands, Queensland, Australia.

### 2.5. Measurements

Participants were scheduled to attend a baseline measurement session (60–90 min) at the University of Newcastle at a time mutually agreeable to them and the researcher. Height and weight were measured, using a standardized protocol, and a fasting blood sample was taken by the researcher and/or a research assistant. These measures, with the exception of height, were repeated at the final measurement session held six weeks after the baseline session. Participants also completed an online questionnaire, either at the session or it was emailed to them to complete later that day. The online questionnaire collected demographic data including age, gender, country of birth, indigenous descent, employment status, and comorbidities. There were also questions to obtain an overall description of pain from participants: cause of pain, main pain site, time experiencing pain, and healthcare use.

The main outcome measures were included in this questionnaire, and participants completed these questions at the baseline and six-week measurement session. These included pain, quality of life, and dietary intake.

#### 2.5.1. Pain

Current pain was measured by participants selecting a point on a 100-mm visual analog scale with a higher score indicating more pain [43]. Overall pain severity (rated on a score of 1–10) and interference (an average of seven items calculated as a score out of 10) were measured using the Brief Pain Inventory, which is a validated pain assessment tool [44,45]. Pain severity was also categorized with a score of 0–4 indicating mild pain, 5–6 moderate pain, and 7–10 severe pain [46]. Pain interference was not categorized, but a higher score denotes higher interference [46]. Pain self-efficacy was measured using the validated Pain Self-Efficacy Questionnaire (PSEQ), which is a sum from 10 questions rated as 0 (not confident at all) to 6 (completely confident) [47]. Results were categorized as severe <20, moderate 20–30, mild 31–40, and minimal impairment >40 [47]. The Pain Catastrophizing Scale (PCS) was used to measure pain catastrophizing with three sub-categories incorporated into the scale: rumination, magnification, and helplessness [48]. A score of <20 indicates mild catastrophizing, 20–30 is high, and >30 is severe. 

#### 2.5.2. Quality of Life

Quality of life was measured using the Short-Form 36 [49] with eight categories containing a number of items including physical function (10 items), role limitations (physical limits) (4 items), role limitations (emotional issues) (3 items), energy and fatigue (4 items), emotional wellbeing (5 items), social functioning (2 items), pain (2 items), and general health (5 items) [49]. Participants gave their responses on a scale from one to three up to one to six depending on the question. This was then scored in ascending or descending order using predetermined values. These were then averaged depending on the number of items, with all questions scored out of 100.

#### 2.5.3. Dietary Intake

This was measured using the Australian Eating Survey Food Frequency Questionnaire (AES FFQ) which aims at capturing typical intake over a long period of time. The AES FFQ also asks how often participants eat 120 commonly consumed foods in Australia, and was validated for use in Australian adults [39]. Upon completion of the AES FFQ, a report was generated which compared the participants’ energy intake and macro- and micro-nutrient breakdown to national dietary guidelines. Diet quality was calculated using the Australian Recommended Food Score (ARFS) [39]. The total score out of 73 was made up of scores from each of the core food groups such as vegetables, fruit, meat and alternatives, grains, dairy, water, and condiments. 

#### 2.5.4. Process Evaluation

Participants’ satisfaction with the program and its components, as well as changes to nutrition-related behaviors, were assessed using the final questionnaire. Participants were asked to rank their satisfaction on a five-point Likert scale for overall satisfaction, AES FFQ satisfaction, juice satisfaction, and for those who received the personalized dietary consultations, the AES report, and telehealth consultations. The response options varied from very satisfied to very unsatisfied. Those participants who received the PDC were asked to rate their agreement that the program encouraged them to change eight nutrition-related behaviors. These nutrition-related behaviors included to eat more fruit and vegetables, eat fewer discretionary choices, change food products they purchased, read nutrition information on food products, keep a record of food and drink consumption, set nutrition goals, download healthy eating apps, and be mindful in using food to cope with pain.

### 2.6. Data Analysis

Data were analyzed using SPSS version 25 (IBM^®^ SPSS^®^ Statistics, IBM Corp. Armonk, NY, USA). Normality testing was undertaken by generating histograms and running the Shapiro–Wilk test to determine if data were normally distributed. Demographics and participants’ description of pain were analyzed using descriptive statistics. Generalized linear mixed models were undertaken for each outcome variable to determine if any effects were due to differences between time (baseline and six weeks), groups (PDC and AFJ; PDC and PFJ; WLC and AFJ; WLC and PFJ), and also group-by-time interaction. Intention to treat was used where there were missing data. Statistical significance was set at *p* < 0.05.

## 3. Results

### 3.1. Number of Study Participants

A total of 391 patients from over 27 HIPS clinical sessions were invited to participate in the study. Of these, 191 returned an expression-of-interest (EOI) form. After screening (*n* = 30 ineligible), 74 participants were eligible and, of these, 60 attended the baseline measurement session and were randomized into the four study arms (Figure 1). The majority of those who did not return an EOI did not participate in follow-up correspondence (phone and/or email) following the HIPS session. At the six-week measurement, 18 participants were lost to follow-up, leaving 42 in the sample (70% retention).

### 3.2. Participant Demographics

Participants were predominantly female (68.3%), with a mean age of 49 ± 15 years and a BMI of 32.6 ± 7.7 kg/m^2^ (Table 1). Ninety percent of participants were born in Australia and seven percent identified as being of Aboriginal and/or Torres Strait Island descent. The most commonly selected employment status was unemployed (due to pain) (*n* = 16), followed by retired (*n* = 12) and part-time paid work (*n* = 10). In terms of self-reported comorbidities, participants reported from zero to five comorbidities with the three most common being depression or anxiety (*n* = 35), osteoarthritis (*n* = 24), and high blood pressure (*n* = 15). There were no significant differences in participant demographics between groups at baseline.

### 3.3. Participants’ Description of Their Pain

The majority of participants (77%) described their pain as always there, but the intensity changes, with the three most commonly reported pain sites being back (*n* = 19), shoulder (*n* = 5), and leg (*n* = 5). Over half the participants (53%) reported they had been experiencing their pain for more than five years, 40% for 1–5 years, and 7% reported having had pain for less than one year. The three most common answers for “how did the main pain begin” included no obvious cause (*n* = 18), related to another illness (*n* = 12), and injury at work or school (*n* = 11). Participants also reported healthcare use in the last three months; on average the participants accessed general practitioners (GPs) about three times and allied health professionals and tests and scans approximately twice in the last three months. Visits to a medical specialist or emergency department for pain were, on average, less than once in the last three months.

### 3.4. Intervention Compliance

Overall, participants reported that they were highly compliant with the intervention protocol, with the majority reporting to have consumed all 42 bottles of juice over the six-week period (Table 2). Only nine participants reported consuming fewer than 42 bottles (ranging from 21–41 bottles), with the main reasons for non-compliance being they forgot or they went on holidays during the six weeks and forgot or were unable to take the juice with them. One participant had to undergo surgery during the study and was only able to consume the juice for 21 days; the data from this participant and all participants were included in the intention-to-treat analysis.

A total of 19 out of 31 participants randomized to the PDC groups took part in the telehealth sessions, completing at least one session. Twelve out of 17 were from the group which also received the AFJ, and seven out of 14 received the PFJ (Table 2). Nine of the participants who did not attend were lost to follow up and also did not attend the second measurement session. The other three participants did attend the final measurement session; however, throughout the duration of the study, they forgot or had other commitments and continually rescheduled their consultations. Participants were required to attend the first two consultations with the third consultation being optional. Of the 19 participants who completed the telehealth sessions, 89% (*n* = 17) attended two or more consultations. The remaining two participants only completed one session.

In the group which received the AFJ, two participants attended one consultation, three participants attended two consultations, and seven participants attended three consultations. In the PFJ group, three participants attended two sessions and four attended three sessions.

### 3.5. Pain Outcomes

When all four groups were compared over time, there was no group-by-time effect for any pain variables. This was also true when the groups were collapsed and all participants receiving PDC were compared to all participants receiving the WLC, and the participants receiving AFJ were compared to those receiving PFJ.

**Figure 2:**
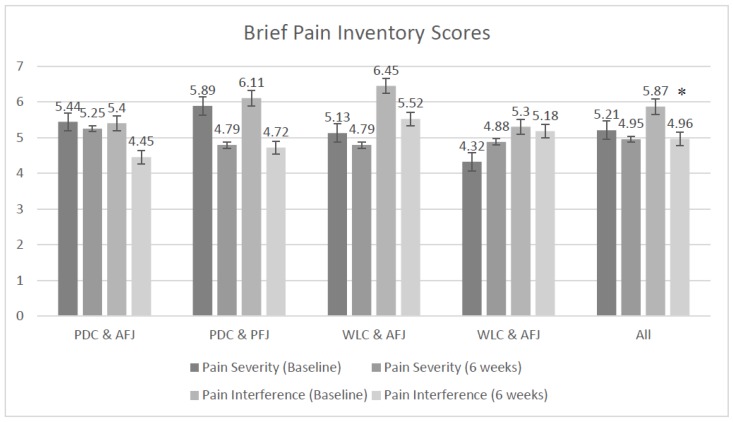
Pain severity and interference (mean ± standard error (SE)). * All groups had a statistically significant reduction in pain interference (*p* = 0.003). PDC + AFJ: personalized dietary consultations and active fruit juice; PDC + PFJ: personalized dietary consultations and placebo fruit juice; WLC + AFJ: waitlist control group and active fruit juice; WLC + PFJ: waitlist control group and placebo fruit juice.

All groups had a statistically significant improvement in three of the five pain variables over the duration of the study (Table 2, Figure 2). These included pain interference (average ∆ −0.9 ± 0.3, *p* = 0.003), pain self-efficacy (average ∆ +6.2 ± 2.2, *p* = 0.004), and pain catastrophizing (average ∆ −3.8 ± 1.8, *p* = 0.046).

The changes between baseline and six weeks for current pain, pain interference, and pain self-efficacy were clinically important, although not statistically significant. Clinical importance is considered as 2.5–3-cm reduction for the visual analog scale measuring current pain [14,50]. Clinically important pain interference is a reduction of >1 point on the BPI inference score and for self-efficacy an increase of ≥7 cm and a change to another severity category on the PSEQ [40]. For current pain, both PDC groups and all groups combined reached clinical importance. Both PDC groups reached clinical importance for pain interference and self-efficacy. Changes in pain severity and pain catastrophizing were not clinically important or statistically significant.

On average, at baseline, all participants rated their pain severity as moderate (BPI) and this did not change over time. Pain self-efficacy was also reported as moderate for all groups at baseline. However, at six weeks, all but the WLC and PFJ groups reported a lower level of pain, and the mean scores were categorized as “mild”. At baseline, all groups and the total were categorized as high for pain catastrophizing; however, at six weeks, all groups, except the PDC and PFJ groups (which remained in the high category), were categorized as mild.

### 3.6. Quality-of-Life Outcomes

The quality-of-life score comprised eight categories. Table 2 indicates that there were no statistically significant differences between groups at baseline. When groups were compared over time, there was no group-by-time effect for any pain variables. This was also true when the results were compared between the PDC group and WLC group and the two supplement groups.

All groups had a statistically significant improvement in six of the eight quality-of-life categories over the duration of the study. These included physical function (average ∆ +8.1 ± 3.4, *p* = 0.016), physical role limitations (average ∆ +20.6 ± 5.6, *p* < 0.001), emotional role limitations (average ∆ +27.1 ± 7.0, *p* < 0.001), emotional wellbeing (average ∆ +8.7 ± 2.8, *p* = 0.003), social functioning (average ∆ +7.4 ± 2.4, *p* = 0.001), and general health (average ∆ +8.3 ± 2.2, *p* < 0.001).

### 3.7. Dietary Outcomes

When groups were compared over time, a significant group-by-time effect was found for reduction in the total percentage energy derived from total fat, with the PDC and AFJ groups achieving a significant reduction in intake (−5.7 ± 2.3%, *p* = 0.024) over time, compared to other groups. This group-by-time effect was also present when the PDC participants (−3.83 ± 1.71%) were compared to the WLC participants (2.06 ± 1.56%), *p* = 0.013. These results were not significant when the AFJ participants (−0.24 ± 1.65%) were compared to the PFJ participants (0.83 ± 1.79%) (*p* = 0.807).

A comprehensive set of food and nutrient-intake and diet-quality variables were evaluated (Table 3). All groups had a statistically significant improvement in three variables over time. These were energy intake (average ∆ −788 ± 364 kJ, *p* = 0.043), percentage energy from core foods (average ∆ +5.2 ± 1.4%, *p* < 0.001) and percentage energy from energy-dense, nutrient-poor foods (average ∆ −5.2 ± 1.4%, *p* < 0.001). Mean energy intake decreased in both PDC groups and the WLC with AFJ (−1540 ± 786 kJ, −652 ± 836, and −1309 ± 689 kJ, respectively) and increased in the WLC and PFJ group (+349 ± 754 kJ).

A description of the participants’ dietary status at baseline shows that the proportion of energy coming from carbohydrates, protein, total fat, saturated fat, and alcohol was 44.4 ± 1.2%, 19.0 ± 0.6%, 34.5 ± 0.9%, 14.8 ± 0.4%, and 2.8 ± 0.7%, respectively. Diet quality, measured using the total ARFS and subcategories, was low at baseline, with the total overall score 29.1 ± 1.4 at baseline. 

### 3.8. Process Evaluation

Overall there were no significant differences between groups in satisfaction and measures obtained within the study process evaluation from participants who completed the study (*n* = 42). The majority of these participants were either satisfied (*n* = 16) or very satisfied (*n* = 19) with the study overall. The remaining participants (*n* = 7) responded as “neutral” when reporting their satisfaction with the study. Overall participants were satisfied or very satisfied with the AES FFQ (*n* = 32), with nine stating they felt neutral and one unsatisfied. Thirty-six participants were satisfied or very satisfied with the fruit juice, with five participants responding as “neutral” and one participant stating they were unsatisfied. 

For those participants who participated in the PDC and who completed the study (*n* = 16 of 42), 100% of participants agreed or strongly agreed that the AES personal nutrition report was useful, helped them identify areas of their diet that could be improved or areas they were already doing well, and provided enough information to guide changes to their dietary intake. Overall, 15 participants were satisfied or very satisfied with the AES report, and one remained neutral. In terms of the dietary consultations, 12 participants were satisfied or very satisfied with four participants remaining neutral. Participants agreed or strongly agreed that being involved in the behavioral intervention encouraged them to consume more fruit and vegetables (100%), less energy-dense, nutrient-poor foods (88%), read nutrition labels (88%), change the food products they commonly purchase (94%), and set nutrition goals (94%).

## 4. Discussion

The aim of the current pilot study was to evaluate whether provision of personalized telehealth dietary consultations with or without supplemental fruit juice high in anthocyanins could lead to a reduction in pain scores within a clinical population experiencing chronic pain. Of all the pain, quality-of-life, and dietary outcomes, the only significant group-by-time effect was for the reduction in total percentage energy from total fat favoring the PDC and AFJ group (−5.7 ± 2.3%, *p* = 0.024). Other significant results involved all groups combined, irrespective of study intervention treatments, where statistically significant improvements were observed in three pain variables, six quality-of-life categories, and three aspects of dietary intake. This pilot study provides a comprehensive description of the change in dietary intake and diet quality compared to the studies identified in the systematic review [12], where few provided a clear description of the intervention and/or a change in these outcomes. The current pilot study provides valuable data on and insight into the feasibility of conducting an evidence-based dietary intervention in a clinical population experiencing chronic pain, and it will be used to inform the design in future trials. 

When comparing the current participants’ demographic and pain information to the Australian and New Zealand dataset consisting of 16790 individuals with chronic pain from the 2016 electronic Persistent Pain Outcomes Collaboration (ePPOC), the current pilot study sample included more women, participants of Aboriginal or Torres Strait Islander descent, and individuals of higher weight status as measured using BMI [53]. Participants in the current pilot study were younger, had a higher BMI, and reported slightly less pain compared to Australian and New Zealand population data; however, all other characteristics were similar between sample populations [53]. These similarities indicate that the participants in this current study are similar to patients attending pain services in Australia and New Zealand, and suggest the results from the current pilot study may be generalizable to the Australian and New Zealand populations experiencing chronic pain. 

Diet quality in the current pilot study was measured by the Australian Recommended Food Score (ARFS), which is a validated brief diet quality index that provides an indication of the relationship between consuming a variety of whole foods and chronic disease risk, with a higher score indicating higher intake and lower risk of disease [54,55]. Low diet quality is linked to an increased risk of cardiovascular disease, some cancers, and higher all-cause mortality [55]. Given that comorbidities are highly prevalent in individuals experiencing chronic pain, more so at HIPS compared to other services [56], it is not surprising that participants reported low diet quality at baseline and six weeks, reflective of limited diet variety overall and also within food group categories. Williams et al. found that the total ARFS among a group of Australian adults (*n* = 93,252) was 34.1 ± 9.7 (vegetable subcategory 11.5 ± 4.3; fruit 5.5 ± 2.9; meat 3.0 ± 1.6; meat alternatives 2.6 ± 1.3; grains 5.7 ± 2.3; dairy 4.0 ± 2.1) [54]. With the exception of the vegetable subcategory, diet quality of participants in the current pilot study was lower than that in the general population and remained low post intervention which is of concern. The vegetable category was comparable between studies with the current pilot study, identifying that, at baseline, the vegetable score was 11.6 ± 0.7 (out of a possible 21) which indicates that, overall, the variety of vegetables usually being consumed over a week in both populations is low. The current pilot study is one of very few to report on diet quality of patients attending a chronic pain service, rather than reporting only selected nutrients [12]. These results suggest that a bigger emphasis should be placed on how to improve overall diet quality and diversity by improving intakes of a range of nutrient-rich foods, and future studies should investigate potential barriers contributing to these poor diet-quality results.

There were significant reductions in total energy intake and percentage energy from energy-dense, nutrient-poor foods, and a significant increase in percentage energy from nutrient-rich core foods in all intervention groups over the duration of the pilot study. The 2011–2012 National Nutrition and Physical Activity Survey (NNPAS) [57] estimated the percentage energy from energy-dense, nutrient-poor foods and macronutrients using a single 24-h recall collected on 12,000 Australian adults [56]. While different dietary assessment tools were used in the NNPAS compared to the current pilot study, it is interesting to compare dietary intakes of the Australian population with the participants in the current pilot study. The NNPAS reported that, in Australia, energy-dense, nutrient-poor foods contribute 35% of total energy intake [57]. At baseline, the participants in this study had 42% of their energy coming from energy-dense, nutrient-poor foods and, after the intervention, this decreased to 37%, which is closer to national data. The distribution of the percentage of energy intake from carbohydrate (NNPAS: 45%, this study: 44%) and protein (18%, 19%) is similar between the two studies [57]. There were differences in total fat, saturated fat, and alcohol with total fat (34%) and saturated fat (15%) higher in this study compared to the NNPAS (31% and 12%, respectively) [57]. The contribution of alcohol to energy intake was lower in this study (2.8%) compared to the national data (3.4%) [57]. From a global perspective, the dietary intake of the participants in this current study can be compared to studies conducted in the United States of America (USA) and Europe. The 2003–2006 National Health and Nutrition Examination Study (NHANES) conducted in the USA collected dietary intake data for 9490 adults using 24-h recall [58]. There were 20 food groups identified as contributing to energy intake [58]. Of these food groups, half could be classified as energy-dense nutrient-poor foods, and approximately 37% of the total energy intake came from these foods [58]. Compared to this current study, the percentage energy coming from energy-dense, nutrient-poor foods was equal after the intervention, where it was calculated that 37% of the energy intake of participants in this current study came from energy-dense, nutrient-poor foods. Total energy intake was higher in the NHANES study (9247 kJ) [58] compared to this current study, where, at baseline, participants were consuming 8870 kJ on average. The distribution of percentage energy from carbohydrates was higher in the USA (52%) compared to 44% in this current study, while the percentage energy from protein was lower (NHANES: 15%, this study: 19%) [58]. Percentage energy from fat intake was the same, with both studies reporting 34%, whereas saturated fat contributed less in the USA (12%) compared to this study (15%) [58]. Total fiber intake was higher in the USA (28 g) compared to this study (22 g) [58]. The European Prospective Investigation into Cancer and Nutrition (EPIC) study also collected dietary intake data using a 24-h recall in 37,000 residents across 10 European countries [59]. On average, the energy intake for males in Europe was higher than the current study with a range of 9223–12083 kJ (compared to 8870 kJ) and lower for females with a range from 6968–8694 kJ (compared to 8870 kJ) [59]. There are vast dietary patterns across Europe which are reflected in the ranges of percentage energy coming from carbohydrates (35–50%), protein (13–21%), and fat (30–42%) [59]. The percentage energy coming from carbohydrates (44%), protein (19%), and fat (34%) at baseline in this current study all fall within these ranges [59]. 

There were two components to the current dietary intervention: the personalized dietary consultations and the dietary supplement. While there was no change to diet quality from the reported intake between groups, the process evaluation shows that it was well accepted. This mirrors results from a recent study which found that nutrition-related goals were reported as being popular among people with chronic pain [50]. While some chronic pain services utilize telehealth to reach patients, it is not used to deliver targeted nutrition advice in a chronic pain population, which makes this a unique pilot study. While only 61% of those randomized to the PDC groups attended any of the consultations, of the 61% who did attend, 89% (*n* = 17) attended the two recommended dietary consultations and/or the third optional consultation. Participants were also highly satisfied with the pilot study and its components. These results suggest that the use of telehealth was acceptable and satisfactory for the delivery of nutrition care to most patients with chronic pain. However, 39% of participants randomized to the PDC groups did not take part in the telehealth intervention component, and further exploration of the reasons behind lack of engagement in this mode of healthcare delivery is warranted. 

The limited between-group differences in the current pilot study may be attributed to the small sample size and potentially a placebo effect such that people enrolling in a dietary intervention, regardless of what intervention they received, had an improvement in pain, quality of life, and dietary intake. Current literature suggests that participants in experimental trials are likely to have a larger placebo effect compared to a clinical trial, and this is particularly so for placebo analgesia research, such as this pilot study, potentially due to the neurotransmitters associated with the placebo response which are shared with the pain experience [60]. In addition, both chronic pain and the placebo effect have a biopsychosocial component. The biomedical aspect involves the neurotransmitters, and both chronic pain and the placebo effect share the same neurotransmitters, which include opioids and dopamine [61]. These are mediated by expectancy, conditioning, motivation, and reward. For example, the expectancy of a reduction in pain can activate the opioid-mediated analgesics pathways, and can lead to an increase in endogenous opioids in those people who experience this. In terms of dopamine, reduced pain can be seen as a reward, and the prospect of pain relief (real or placebo) can stimulate the dopaminergic pathway leading to a reduction in the chronic pain experience. These responses are also modulated by the context or the psychosocial aspects of receiving a placebo treatment. The relationship between the participant and the researcher also influences the placebo response. Attending a medical specialist appointment or participating in a research study triggers a response before any treatment is given. Studies showed that a placebo treatment is effective in people with chronic pain [61,62,63]. It is possible, in this pilot study, that the participants had high expectations, especially given the novel nature of the treatment. Anecdotally, many participants expressed that they were willing to “try anything” to help relieve their pain. The current pilot study was also delivered by a qualified expert in nutrition and dietetics, and supported by clinicians from a tertiary pain service; hence, the experience and credibility of the research team may have influenced the participants’ expectations coming into the study and, therefore, contributed to a placebo response [60,61].

This current pilot study found clinically important results favoring the PDC group relative to the WLC group. Clinically important changes for the BPI, PSEQ, and PCS were established through the establishment of ePPOC [46]. For pain severity (BPI), minimal, moderate, and substantial improvement is classified as an increase by 10%, 30%, or 50%, respectively, with emphasis on moderate and substantial change [46]. In the current pilot study, only the PDC and PFJ group reached minimal clinically important improvements. Clinically important change for pain interference (BPI) and self-efficacy (PSEQ) was achieved in both PDC groups, as demonstrated by at least a one-point reduction in the BPI score and seven or more point increase, with movement to a lower pain severity category in the PSEQ [46]. There were no clinically important results for the PCS. In terms of the visual analog scale (VAS), there are mixed reports with regards to what is considered clinically important, with some studies reporting a 2.5-cm change and others reporting a 3-cm change as considered clinically important [14,50]. In the current pilot study, there was a clinically important group effect when all groups were combined, with a >3-cm decrease in pain as measured by PDC groups having the highest change compared to the WLC groups with a maximum 2-cm reduction. These results indicate that the treatment effect for people experiencing chronic pain is of a sufficient magnitude to warrant clinical pain services to consider including a personalized dietary intervention into their treatment program to further improve the pain experience of their patients. In the current pilot study, 52% of participants had clinically important changes in pain interference and pain self-efficacy. This change can be compared to data reported by ePPOC that 68% of patients had clinical important improvements in pain interference and 48% in pain self-efficacy [53]. Two studies examined the effect of the use of amitriptyline in managing pain triggered by spinal cord injury [64] or amputation [65]. These studies did not find any statistically significant results post intervention [64,65]. However, when the data were pooled and examined for clinical importance, approximately one-third (33%) of participants reported a clinically meaningful reduction in pain, as measured using a self-reported pain rating scale [66]. Similarly, a study exploring a self-help intervention based on acceptance and commitment therapy found that 28% of those in the intervention group had a clinically important benefit for pain interference [67]. The proportion of participants who had a clinically meaningful reduction in pain was lower in both of these studies, compared to this current pilot study and multidisciplinary care (as shown in the ePPOC data). The difference between these studies and the current pilot study is that the use of medication is a passive treatment, while self-help provides personalization or guidance to assist participants. 

One of the limitations of this study was the high loss to follow-up with 30% of participants dropping out of the study, with a higher proportion coming from the treatment groups than the control groups. In addition, of those who were randomized to receive the personalized dietary consultations, only 61% completed at least one session. It is possible that only those who perceived a benefit from the study decided to complete the study. The small sample size and lower power to determine statistically significant outcomes were other limitations of the current study. However, this was a pilot study to determine whether the inclusion of a telehealth component for delivery of personalized dietary counselling by a dietitian is acceptable, and to assess the preliminary impact over a relatively short duration. The use of apple juice as the control comparator was also a potential limitation. Despite the low anthocyanin and flavonoid content, there is the potential that participants may have received benefit from this juice. In addition, previous research using apple juice as a control comparator involved cognition studies; further research is needed to see if this applies to pain management. The current study demonstrates the feasibility of this technology in delivering nutrition care to this group. Many individuals with chronic pain have additional barriers to attending physical in-person sessions with a dietitian. As such, this further supports the need for a larger, appropriately powered trial to determine effectiveness. 

To the authors’ knowledge, this is the first pilot study to implement a comprehensive dietary intervention of high methodological quality within a chronic pain clinical population. The other strengths of the current trial should be acknowledged, and include the stratification of males and females as part of the randomization process. The current pilot study also addressed some of the limitations of current literature exploring nutrition’s role in pain management [12], such as providing a clear description of the dietary intervention used, compared to previous studies of the effect of nutrition in pain management, which had poor descriptions. Furthermore, validated measures to assess pain, quality of life, and dietary intake were used. Previous studies often relied on a single item measure, such as a VAS, which is easy to implement but does not capture the complexity of pain. Other tools such as the BPI, PSEQ, and PCS incorporate the biopsychosocial factors involved in the pain experience and provide a multidimensional measurement of pain. The current pilot study included all these measures so that results could be more easily compared to other studies which used a VAS, but also to provide a robust assessment of pain using multidimensional tools. The current pilot study also included a validated FFQ to accurately evaluate participants’ dietary intake and diet quality. Providing a detailed description of methods and intervention allows replication and future refinements.

## 5. Conclusions

While group-by-time differences were not statistically significant, all groups demonstrated improvements in perceived pain, quality of life, and dietary intake. Improvements in pain interference and pain self-efficacy were clinically meaningful in the two groups receiving personalized dietary consultations compared to the waitlist control groups. The current pilot study demonstrates potential benefits from providing people who experience chronic pain with a personalized dietary intervention using telehealth. The current pilot study provides data to inform sample size calculations for a future multicenter trial to determine the efficacy of a personalized dietary intervention as part of chronic pain management. Future studies should also consider potential motivators and barriers which may have contributed to the results of the current pilot study to improve the trial design and success of future studies.

## Figures and Tables

**Table 1:** Demographic characteristics of participants at baseline.

	PDC + AFJ (*n* = 17)	PDC + PFJ (*n* = 14)	WLC + AFJ (*n* = 15)	WLC + PFJ (*n* = 14)	Total (*n* = 60)	*p*-Value
Female *n* (%)	12 (70.6)	10 (71.4)	9 (60)	10 (71.4)	41 (68.3)	0.896
Male *n* (%)	5 (29.4)	4 (28.6)	6 (40)	4 (28.6)	19 (31.7)	
Age	48.24 ± 14.60	47.00 ± 15.66	49.27 ± 16.72	50.93 ± 13.85	48.83 ± 14.92	0.930
BMI	33.14 ± 8.31	32.83 ± 8.15	33.43 ± 5.82	30.78 ± 8.74	32.59 ± 7.70	0.561
Employment *n* (%)						0.088
- Unemployed (due to pain)	7 (41.2)	4 (28.6)	5 (33.3)	0 (0)	16 (26.7)	
- Retired	3 (17.6)	2 (14.3)	4 (26.7)	3 (21.4)	12 (20)	
- Part-time paid work	0 (0)	3 (21.4)	3 (20.0)	4 (28.6)	10 (16.7)	
- Unemployed (not due to pain)	0 (0)	2 (14.3)	0 (0)	3 (21.4)	5 (8.3)	
- Home duties	1 (5.9)	2 (14.3)	0 (0)	1 (7.1)	4 (6.7)	
- Full-time paid work	2 (11.8)	1 (7.1)	0 (0)	1 (7.1)	4 (6.7)	
- Studying	2 (11.8)	0 (0)	2 (13.3)	0 (0)	4 (6.7)	
- At work (limited hours/duties)	1 (5.9)	0 (0)	1 (6.7)	1 (7.1)	3 (5.0)	
- On leave from work due to pain	1 (5.9)	0 (0)	0 (0)	1 (7.1)	2 (3.3)	

PDC + AFJ: personalized dietary consultations and active fruit juice; PDC + PFJ: personalized dietary consultations and placebo fruit juice; WLC + AFJ: waitlist control group and active fruit juice; WLC + PFJ: waitlist control group and placebo fruit juice. The *p*-values were calculated using ANOVA and exact chi-squared tests.

**Table 2:** Pain and quality-of-life outcomes.

Outcome Variable (mean ± SE ^*^)	Time Point	PDC + AFJ	PDC + PFJ	WLC + AFJ	WLC + PFJ	Total	Time F Stat (*p*-Value)	Group F Stat (*p*-Value)	Group × Time F Stat (*p*-Value)
Number of participants	Baseline	17	14	15	14	60			
	6 weeks	11	8	13	10	42			
Number of juice bottles consumed (Maximum 42)	6 weeks	39.64 ± 1.89	41.00 ± 0.76	41.46 ± 0.31	41.90 ± 0.10	41.00 ± 3.39			
Telehealth attendance	N/A	12	7	N/A	N/A	N/A			
Pain									
VAS ^†^	Baseline	48.24 ± 5.31	52.00 ± 5.85	45.40 ± 5.65	47.64 ± 5.85	48.27 ± 2.75	1.16 (0.254)	0.102 (0.959)	0.113 (0.952)
	6 weeks	43.87 ± 6.39	44.99 ± 7.43	43.61 ± 6.00	45.68 ± 6.74	44.57 ± 3.20			
	∆6 week − baseline	−4.37 ± 6.71	−7.02 ± 7.77	−1.78 ± 6.39	−1.96 ± 7.11	−3.69 ± 3.37			
PSEQ ^^^	Baseline	26.82 ± 3.07	21.29 ± 3.38	26.87 ± 3.27	25.21 ± 3.38	25.17 ± 1.62	8.835 **(0.004)**	0.606 (0.613)	1.181 (0.321)
	6 weeks	35.21 ± 3.74	33.61 ± 4.37	30.75 ± 3.48	26.41 ± 3.94	31.37 ± 1.91			
	∆6 week − baseline	8.38 ± 4.15	12.32 ± 4.79	3.89 ± 3.67	1.19 ± 4.40	6.21 ± 2.16			
PCS ^#^	Baseline	21.24 ± 3.27	27.43 ± 3.60	21.60 ± 3.48	24.93 ± 3.60	23.63 ± 1.72	4.074 **(0.046)**	0.831 (0.480)	0.073 (0.974)
	6 weeks	18.23 ± 3.80	24.70 ± 4.37	17.63 ± 3.64	19.96 ± 4.03	19.86 ± 1.93			
	∆6 week − baseline	−3.02 ± 3.49	−2.73 ± 4.05	−3.97 ± 3.28	−4.97 ± 3.68	−3.78 ± 1.75			
Quality of life									
Physical function	Baseline	45.59 ± 6.91	42.86 ± 7.62	36.67 ± 7.36	38.57 ± 7.62	41.08 ± 3.63	6.040 **(0.016)**	0.689 (0.561)	0.293 (0.830)
	6 weeks	55.86 ± 7.87	56.22 ± 9.04	42.25 ± 7.66	43.79 ± 8.41	49.13 ± 4.02			
	∆6 week − baseline	10.28 ± 6.72	13.37 ± 7.83	5.58 ± 6.30	5.22 ± 7.09	8.05 ± 3.38			
Role limitation (physical limits)	Baseline	23.53 ± 7.97	12.50 ± 8.78	1.67 ± 8.48	10.71 ± 8.78	12.50 ± 4.28	14.133 **(<0.001)**	2.053 (0.112)	0.238 (0.870)
	6 weeks	43.78 ± 9.76	34.78 ± 11.40	17.23 ± 9.06	39.48 ± 10.26	33.13 ± 5.03			
	∆6 week − baseline	20.25 ± 11.04	22.28 ± 12.74	15.57 ± 10.59	28.77 ± 11.73	20.63 ± 5.62			
Role limitation (emotional issues)	Baseline	50.98 ± 9.78	23.81 ± 10.77	26.67 ± 10.41	19.05 ± 10.77	31.11 ± 5.23	16.526 **(<0.001)**	0.369 (0.776)	1.838 (0.146)
	6 weeks	53.41 ± 11.93	60.98 ± 13.94	55.10 ± 11.10	64.24 ± 12.56	58.25 ± 6.17			
	∆6 week − baseline	2.43 ± 13.31	37.17 ± 15.37	28.44 ± 12.75	45.19 ± 14.14	27.14 ± 7.02			
Energy & fatigue	Baseline	35.29 ± 4.84	33.21 ± 5.34	22.67 ± 5.16	30.71 ± 5.34	30.58 ± 2.62	4.650 (0.34)	2.409 (0.072)	0.639 (0.592)
	6 weeks	48.56 ± 5.75	35.67 ± 6.67	26.95 ± 5.45	26.63 ± 6.09	39.92 ± 3.00			
	∆6 week − baseline	13.26 ± 5.75	2.46 ± 6.68	4.28 ± 5.45	5.92 ± 6.09	6.33 ± 2.97			
Emotional wellbeing	Baseline	60.71 ± 4.79	49.43 ± 5.28	54.40 ± 5.10	50.29 ± 5.28	54.07 ± 2.53	9.348 **(0.003)**	0.488 (0.692)	0.485 (0.693)
	6 weeks	63.81 ± 5.67	61.86 ± 6.57	64.25 ± 5.38	60.76 ±6.00	62.80 ± 2.89			
	∆6 week **−** baseline	3.12 ± 5.62	12.43 ± 6.52	9.85 ± 5.32	10.48 ± 5.94	8.73 ± 2.83			
Social functioning	Baseline	27.94 ± 3.24	25.89 ± 3.58	16.67 ± 3.45	15.18 ± 3.58	21.67 ± 1.84	11.342 **(0.001)**	5.285 (0.002)	0.401 (0.753)
	6 weeks	37.15 ± 3.98	33.36 ± 4.66	20.98 ± 3.69	26.30 ± 4.19	29.07 ± 2.16			
	∆6 week **−** baseline	9.21 ± 4.55	7.46 ± 5.25	4.32 ± 4.38	11.12 ± 4.84	7.40 ± 2.36			
Pain	Baseline	37.21 ± 4.83	48.39 ± 5.33	36.00 ± 5.15	36.96 ± 5.33	39.46 ± 2.57	0.345 (0.559)	1.107 (0.350)	0.768 (0.515)
	6 weeks	44.91 ± 5.95	44.02 ± 6.96	33.65 ± 5.51	44.49 ± 6.25	41.27 ± 3.05			
	∆6 week **−** baseline	7.71 ± 6.92	−4.38 ± 7.97	−2.35 ± 6.66	7.52 ± 7.36	1.82 ± 3.59			
General health	Baseline	43.82 ± 4.82	38.93 ± 5.31	39.67 ± 5.13	27.86 ± 5.31	37.92 ± 2.65	15.839 **(<0.001)**	2.567 (0.059)	0.958 (0.416)
	6 weeks	54.10 ± 5.40	53.59 ± 6.17	44.30 ± 5.31	33.98 ± 5.78	46.17 ± 2.89			
	∆6 week **−** baseline	10.27 ± 4.30	14.66 ± 5.02	4.63 ± 4.02	6.12 ± 4.54	8.25 ± 2.19			

PDC + AFJ: personalized dietary consultations and active fruit juice; PDC + PFJ: personalized dietary consultations and placebo fruit juice; WLC + AFJ: waitlist control group and active fruit juice; WLC + PFJ: waitlist control group and placebo fruit juice. * Standard error; † visual analog scale; ^ Pain Self-Efficacy Questionnaire; # Pain Catastrophizing Scale; N/A: not available. Significant *p*-values (<0.05) have been highlighted throughout the table.

**Table 3:** Dietary outcomes.

Outcome Variable (mean ± SE ^*^)	Time Point	PDC + AFJ	PDC + PFJ	WLC + AFJ	WLC + PFJ	Total	Time F Stat (*p*-Value)	Group F Stat (*p*-Value)	Group × Time F Stat (*p*-Value)
Energy (kJ) (recommended intake)	Baseline	9247.00 ± 932.65	9051.39 ± 1034.68	9046.07 ± 963.23	8138.00 ± 1034.98	8870.61 ± 496.16	4.210 (0.043)	0.032 (0.992)	1.263 (0.292)
	6 weeks	7708.05 ± 1059.79	8399.67 ± 1149.39	7736.72 ± 1007.31	8487.45 ± 1094.25	8082.97 ± 239.08			
	∆6 week **−** baseline	−1539.92 ± 785.52	−651.72 ± 835.58	−1309.35 ± 689.08	349.45 ± 753.60	−787.64 ± 363.89			
Carbohydrates (% of total energy (E)) 45–65% [51]	Baseline	43.06 ± 2.32	46.31 ± 2.58	42.87 ± 2.40	45.31 ± 2.58	44.39 ± 1.24	1.703 (0.195)	1.530 (0.212)	0.440 (0.725)
	6 weeks	40.33 ± 3.01	46.06 ± 3.21	38.04 ± 2.65	44.61 ± 2.90	42.26 ± 1.47			
	∆6 week **−** baseline	−2.74 ± 2.29	−0.25 ± 3.52	−4.82 ± 2.97	−0.70 ± 3.24	−2.13 ± 1.63			
Protein (% total E) 15–25% [51]	Baseline	19.81 ± 1.08	18.23 ± 1.20	21.00 ± 1.12	17.08 ± 1.20	19.03 ± 0.58	0.128 (0.721)	2.920 (0.038)	0.769 (0.515)
	6 weeks	18.50 ± 1.35	18.38 ± 1.44	22.22 ± 1.21	17.95 ± 1.32	19.26 ± 0.67			
	∆6 week **−** baseline	−1.31 ± 1.32	0.15 ± 1.41	1.21 ± 1.18	0.88 ± 1.28	0.23 ± 0.65			
Fat (% total E) 20–35% [51]	Baseline	35.81 ± 1.61	33.69 ± 1.79	35.20 ± 1.66	33.08 ± 1.79	34.45 ± 0.86	0.633 (0.428)	2.156 (0.099)	3.290 **(0.024)**
	6 weeks	30.09 ± 2.10	32.07 ± 2.23	39.13 ± 1.84	32.87 ± 2.01	33.54 ± 1.02			
	∆6 week **−** baseline	−5.73 ± 2.30 **	−1.62 ± 2.47	3.93 ± 2.09	−0.21 ± 2.27	−0.91 ± 1.14			
Saturated fat (% total E) <10% [52]	Baseline	14.63 ± 0.83	14.46 ± 0.92	15.33 ± 0.86	14.85 ± 0.92	14.82 ± 0.44	1.624 (0.206)	2.259 (0.087)	2.029 (0.116)
	6 weeks	11.88 ± 1.08	13.70 ± 1.15	16.50 ± 0.95	14.21 ± 1.03	14.07 ± 0.53			
	∆6 week **−** baseline	−2.75 ± 1.18	−0.77 ± 1.26	1.17 ± 10.7	−0.63 ± 1.16	−0.75 ± 0.59			
Alcohol (% total E) <5% [52]	Baseline	2.00 ± 1.26	2.39 ± 1.39	1.47 ± 1.30	5.23 ± 1.39	2.77 ± 0.67	0.034 (0.854)	2.485 (0.066)	1.541 (0.209)
	6 weeks	0.04 ± 1.46	4.03 ± 1.58	1.06 ± 1.37	5.52 ± 1.49	2.66 ± 0.74			
	∆6 week **−** baseline	-1.96 ± 1.18	1.65 ± 1.26	−0.40 ± 1.04	0.29 ± 1.14	−0.12 ± 0.18			
Saturated fat (% total fat)	Baseline	44.81 ± 1.60	47.31 ± 1.77	47.40±1.65	48.54 ± 1.77	47.02 ± 0.85	3.085 (0.083)	3.840 (0.012)	1.054 (0.373)
	6 weeks	38.85 ± 2.12	46.98 ± 2.25	46.20 ± 1.84	47.34 ± 2.01	44.84 ± 1.03			
	∆6 week **−** baseline	−5.97 ± 2.48	−0.32 ± 2.66	−1.20 ± 2.27	−1.20 ± 2.47	−2.17 ± 1.24			
MUFA (% total fat)	Baseline	41.56 ± 1.28	40.31 ± 1.42	40.13 ± 1.32	39.54 ± 1.42	40.39 ± 0.68	0.109 (0.742)	0.208 (0.891)	1.444 (0.236)
	6 weeks	37.80 ± 1.69	40.41 ± 1.80	41.55 ± 1.47	40.47 ± 1.61	40.06 ± 0.82			
	∆6 week **−** baseline	−3.76 ± 1.99	0.10 ± 2.14	1.42 ± 1.83	0.94 ± 1.99	−0.33 ± 0.99			
PUFA (% total fat)	Baseline	41.56 ± 1.28	40.31 ± 1.42	40.13 ± 1.32	39.54 ± 1.42	40.39 ± 0.68	0.109 (0.742)	0.208 (0.891)	1.444 (0.236)
	6 weeks	37.80 ± 1.69	40.41 ± 1.80	41.55 ± 1.47	40.47 ± 1.61	40.06 ± 0.82			
	∆6 week **−** baseline	−3.76 ± 1.99	0.10 ± 2.14	1.42 ± 1.83	0.94 ± 1.99	−0.33 ± 0.99			
Fiber (g) 25–30 g/day [51]	Baseline	25.79 ± 2.41	25.17 ± 2.67	22.79 ± 2.49	19.71 ± 2.67	23.39 ± 1.28	2.365 (0.128)	0.901 (0.444)	0.593 (0.621)
	6 weeks	23.54 ± 2.86	23.19 ± 3.08	19.15 ± 2.65	20.24 ± 2.88	21.53 ± 1.44			
	∆6 week **−** baseline	−2.25 ± 2.47	−1.98 ± 2.67	−3.74 ± 2.18	0.54 ± 2.38	−1.86 ± 1.21			
Thiamin (mg)	Baseline	1.53 ± 0.18	1.60 ± 0.20	1.47 ± 0.19	1.36 ± 0.20	1.49 ± 0.10	1.288 (0.260)	0.398 (0.755)	0.432 (0.730)
	6 weeks	1.43 ± 0.21	1.57 ± 0.23	1.21 ± 0.20	1.34 ± 0.22	1.39 ± 0.12			
	∆6 week **−** baseline	-0.10 ± 0.18	−0.03 ± 0.20	−0.26 ± 0.16	−0.02 ± 0.18	−0.10 ± 0.09			
Riboflavin (mg)	Baseline	2.19 ± 0.35	2.40 ± 0.38	2.56 ± 0.36	1.80 ± 0.38	2.24 ± 0.18	0.247 (0.620)	0.602 (0.615)	0.341 (0.796)
	6 weeks	2.18 ± 0.37	2.38 ± 0.41	2.34 ± 0.37	1.84 ± 0.40	2.18 ± 0.19			
	∆6 week **−** baseline	−0.02 ± 0.23	−0.01 ± 0.24	−0.23 ± 0.20	0.04 ± 0.22	−0.06 ± 0.11			
Niacin (mg)	Baseline	26.99 ± 2.60	23.693 ± 2.88	24.16 ± 2.68	20.70 ± 2.88	23.87 ± 1.38	0.703 (0.404)	0.358 (0.783)	0.948 (0.421)
	6 weeks	23.62 ± 3.05	23.73 ± 3.29	21.53 ± 2.84	22.44 ± 3.09	22.83 ± 1.54			
	∆6 week **−** baseline	−3.37 ± 2.53	0.10 ± 2.69	−2.63 ± 2.23	1.75 ± 2.44	−1.04 ± 1.24			
Calcium (mg)	Baseline	971.25 ± 226.83	1160.92 ± 251.65	1483.19 ± 234.27	827.11 ± 251.65	1110.62 ± 120.63	0.340 (0.562)	1.307 (0.277)	0.256 (0.857)
	6 weeks	972.89 ± 238.22	1051.28 ± 261.85	1445.13 ± 238.12	847.12 ± 256.59	1079.10 ± 124.46			
	∆6 week **−** baseline	1.63 ± 111.10	−109.64 ± 117.95	−38.06 ± 96.61	20.01 ± 105.78	−31.51 ± 54.07			
Iron (mg)	Baseline	13.47 ± 1.46	13.06 ± 1.62	12.75 ± 1.51	11.22 ± 1.62	12.63 ± 0.78	1.545 (0.217)	0.156 (0.925)	1.978 (0.123)
	6 weeks	11.40 ± 1.62	13.16 ± 1.77	11.04 ± 1.56	12.26 ± 1.69	11.96 ± 0.83			
	∆6 week **−** baseline	−2.08 ± 1.09	0.10 ± 1.16	−1.71 ± 0.96	1.04 ± 1.04	−0.66 ± 0.53			
Zinc (mg)	Baseline	13.69 ± 1.56	12.65 ± 1.73	14.48 ± 1.61	11.02 ± 1.73	12.96 ± 0.83	1.410 (0.238)	0.344 (0.793)	1.506 (0.219)
	6 weeks	11.68 ± 1.74	11.72 ± 1.89	13.27 ± 1.67	12.36 ± 1.81	12.26 ± 0.89			
	∆6 week **−** baseline	−2.01 ± 1.21	−0.93 ± 1.29	−1.20 ± 1.06	1.34 ± 1.16	0.70 ± 0.59			
Core (%E)	Baseline	57.77 ± 3.31	57.77 ± 3.78	67.20 ± 3.52	47.92 ± 3.78	57.66 ± 1.80	13.286 **(<0.001)**	6.186 (0.001)	0.633 (0.596)
	6 weeks	61.88 ± 3.94	61.67 ± 4.20	75.52 ± 3.68	52.34 ± 3.99	52.85 ± 1.98			
	∆6 week **−** baseline	4.11 ± 3.03	3.90 ± 3.05	8.32 ± 2.52	4.42 ± 2.75	5.19 ± 1.42			
Energy-dense, nutrient-poor (%E)	Baseline	42.24 ± 3.21	42.23 ± 3.78	32.80 ± 3.52	52.08 ± 3.78	42.34 ± 1.80	13.286 **(<0.001)**	6.186 (0.001)	0.633 (0.596)
	6 weeks	38.12 ± 3.94	38.33 ± 4.20	24.48 ± 3.68	47.66 ± 3.99	37.15 ± 1.98			
	∆6 week **−** baseline	−4.11 ± 3.03	−3.90 ± 3.05	−8.23 ± 2.52	−4.42 ± 2.75	−5.19 ± 1.42			
ARFS ^†^: Total (73 points)	Baseline	30.69 ± 2.65	32.54 ± 2.94	27.60 ± 2.74	25.69 ± 2.94	29.13 ± 1.41	0.028 (0.868)	1.080 (0.362)	0.211 (0.888)
	6 weeks	31.49 ± 3.06	31.95 ± 3.23	26.64 ± 2.85	26.82 ± 3.08	29.30 ± 1.53			
	∆6 week **−** baseline	0.81 ± 2.21	−0.59 ± 2.22	−0.66 ± 1.83	1.13 ± 2.00	0.17 ± 1.04			
ARFS: Vegetables (21 points)	Baseline	13.31 ± 1.33	11.77 ± 1.48	10.20 ± 1.37	10.92 ± 1.48	11.55 ± 0.71	0.400 (0.529)	0.819 (0.487)	0.259 (0.855)
	6 weeks	12.77 ± 1.67	13.10 ± 1.72	10.64 ± 1.47	11.49 ± 1.60	12.00 ± 0.81			
	∆6 week **−** baseline	−0.54 ± 1.50	1.33 ± 1.52	0.44 ± 1.26	0.56±1.38	0.45 ± 0.71			
ARFS: Fruit (12 points)	Baseline	3.44 ± 0.77	6.08 ± 0.85	4.73 ± 0.79	2.77±0.85	4.25 ± 0.41	0.569 (0.453)	2.153 (0.099)	0.863 (0.464)
	6 weeks	3.70 ± 0.90	4.92 ± 0.94	4.62 ± 0.83	2.82±0.90	4.01 ± 0.45			
	∆6 week **−** baseline	0.26 ± 0.68	−1.15 ± 0.68	−0.11 ± 0.56	0.05±0.62	−0.24 ± 0.32			
ARFS: Meat, chicken & fish (7 points)	Baseline	2.81 ± 0.35	3.15 ± 0.39	2.40 ± 0.36	2.31±0.39	2.67 ± 0.19	0.064 (0.801)	1.090 (0.358)	0.107 (0.956)
	6 weeks	3.03 ± 0.46	3.01 ± 0.47	2.45 ± 0.39	2.40±0.43	2.72 ± 0.22			
	∆6 week **−** baseline	0.22 ± 0.45	−0.14 ± 0.46	0.05 ± 0.39	0.10±0.42	0.06 ± 0.22			
ARFS: Vegetarian choices (6 or 12 points)	Baseline	2.13 ± 0.31	1.85 ± 0.34	1.47 ± 0.32	1.54±0.34	1.74 ± 0.17	1.663 (0.201)	0.940 (0.425)	0.160 (0.923)
	6 weeks	1.72 ± 0.42	1.74 ± 0.42	1.33 ± 0.35	1.13±0.38	1.48 ± 0.20			
	∆6 week **−** baseline	−0.41 ± 0.43	−0.11 ± 0.44	−0.14 ± 0.37	−0.41 ± 0.40	−0.27 ± 0.21			
ARFS: Grains (13 points)	Baseline	3.94 ± 0.47	3.92 ± 0.52	4.07 ± 0.48	3.54 ± 0.52	3.87 ± 0.25	1.189 (0.279)	0.182 (0.908)	1.350 (0.264)
	6 weeks	4.41 ± 0.61	4.44 ± 0.62	3.50 ± 0.52	4.32 ± 0.57	4.17 ± 0.29			
	∆6 week **−** baseline	0.47 ± 0.58	0.52 ± 0.59	−0.57 ± 0.49	0.78 ± 0.54	0.30 ± 0.28			
ARFS: Dairy (11 points)	Baseline	3.38 ± 0.46	4.62 ± 0.51	3.27 ± 0.47	3.54 ± 0.51	3.70 ± 0.24	0.363 (0.548)	0.782 (0.507)	0.959 (0.416)
	6 weeks	3.67 ± 0.57	3.79 ± 0.59	3.15 ± 0.50	3.63 ± 0.55	3.56 ± 0.28			
	∆6 week **−** baseline	0.29 ± 0.50	−0.83 ± 0.50	−0.12 ± 0.42	0.09 ± 0.46	−0.14 ± 0.23			
ARFS: Condiments (2 points)	Baseline	1.19 ± 0.18	0.62 ± 0.20	0.73 ± 0.19	0.62 ± 0.20	0.79 ± 0.10	1.460 (0.230)	3.374 (0.022)	1.115 (0.347)
	6 weeks	1.46 ± 0.24	0.86 ± 0.24	0.53 ± 0.20	0.86 ± 0.22	0.93 ± 0.11			
	∆6 week **−** baseline	0.27 ± 0.24	0.25 ± 0.25	0.20 ± 0.21	0.25 ± 0.23	0.14 ± 0.12			
ARFS: Water (1 point)	Baseline	0.50 ± 0.12	0.54 ± 0.14	0.73 ± 0.13	0.46 ± 0.14	0.56 ± 0.07	1.138 (0.254)	1.867 (0.141)	1.911 (0.134)
	6 weeks	0.75 ± 0.15	0.61 ± 0.16	0.85 ± 0.13	0.30 ± 0.15	0.63 ± 0.07			
	∆6 week **−** baseline	0.25 ± 0.13	0.08 ± 0.13	0.11 ± 0.11	−0.16 ± 0.12	0.07 ± 0.06			

PDC + AFJ: personalized dietary consultations and active fruit juice; PDC + PFJ personalized dietary consultations and placebo fruit juice; WLC + AFJ: waitlist control group and active fruit juice; WLC + PFJ: waitlist control group and placebo fruit juice. * Standard error; † Australian Recommended Food Score; ** statistically significant; MUFA: monounsaturated fatty acid; PUFA: polyunsaturated fatty acid. Significant *p*-values (<0.05) have been highlighted throughout the table.

## Supplementary Materials

The following are available online at http://www.mdpi.com/2072-6643/11/1/181/s1: Table S1: Nutrition information for fruit juices.
