# Supplementary material for: The Effect of a Pilot Dietary Intervention on Pain Outcomes in Patients Attending a Tertiary Pain Service
Source: Nutrients. 2019 Jan 16;11(1):181. doi: 10.3390/nu11010181 (PMC6357136; doi:10.3390/nu11010181)
Supplement: Supplementary file 1 [file nutrients-11-00181-s001.pdf]

Table S1. Nutrition information for fruit juices.

	Cherry crush (per 100 mL)	Apple juice (per 100 mL)
Energy (kJ)	291	185
Protein (g)	1.6	0.1
Fat (total) (g)	<0.2	0.0
Carbohydrates (g)	14.7	12.0
Sugars (g)	14.7	11.7
Dietary fibre (g)	0.8	0.1
Sodium (mg)	<1	5
Vitamin C (mg)	<5	40
Total red count (mg/100g)	19.3	0
